# A Plea for Conservatism in Gynecology

**Published:** 1893-05

**Authors:** D. R. Wallace

**Affiliations:** Waco, Texas


For Daniel's Texas Medical Journal.

### A PLEA FOR CONSERVATISM IN GYNECOLOGY.

D. R. WALLACE, M. D. LL. D., WACO, TEXAS.

[Read at Fort Worth meeting North Texas Medical Society, 1893.]

GYNECOLOGICAL surgery may be said to have had its origin within living memory. Still confined within the narrow limits of a few decades, its practice is now co-extensive with the civilized world. It is no part of the purpose of this hasty paper to attempt an exhaustive treatment of the subject. The field along all of its lines and phases is much too broad.

The laws of human progress, as the greatest of living philosophers has exhaustively shown and abundantly illustrated, like those of the material universe, are rythmic in their action. Action; then come re-action. The pendulum of advance goes to an extreme limit to-day, only to traverse a like arc to-morrow, until at last the limit is reached which the data and conditions of the age fix as the limit of advance for the time being. Our profession can constitute no exception, as the law is universal. We may example the familiar history of some of the most useful articles of the pharmacopœia. It is believed there is not a single one, however simple in its action and beneficient in result, or however popular, but has suffered eclipse, fallen into desuetude, and, perhaps, been banished from use for a time. Instance, Peruvian bark, tartarized antimony, mercury, opium, and in certain places and schools, even chloroform, chloral, the bromides, and the most useful of the coal-tar preparations. Surgery more palpable in its methods and technique, and obvious in result to inspection, is subject, though in a less degree, to similar waves of advance and retreat in professional estimation.

For reasons, some of which are obvious, gynecological surgery up to within recent times, did not keep pace with the general science. Its whole armamentarium, until the last century or two, consisted of the uterine sound, sponge-tents and the dioptra. Medical science seems to have made considerable advance among the Egyptians prior to the age of Herodotus, who tells us that in his travels through this wonder-land he found physicians everywhere, each physician applying himself to one disease, and no more,—some for the eye, others for the head, others for the teeth and others for internal diseases. In time of the Ptolemys dissection of the human body, permitted so far as known by no other people of antiquity, was legalized. Still, though general surgery was considerably advanced, we read of none in connection with the pelvic viscera of the female.

Soranus, though he wrote a history of medicine and a biography of Hippocrates, and a work entitled ":De Utero et Pudendo Muliebri," does not mention any surgical procedure connected with these parts.

Topical treatment did not go beyond the use of astringents,

---

NOTE.—Dr. H. W. Brown, of Waco, ex-President Texas State Medical Association, and an old practitioner, on hearing this paper read, said "Doctor Wallace, I want you to have that paper published; it will do good. I did, in early life, a leading gynecological practice in Griffin, Ga., afterwards in Atlanta,—and you know what it has been for the last twenty-five years in Waco, and I pledge you my word, I have never had occasion to perform a laparotomy. I have had cases in which I thought I would have to; but, by drawing off water and making persistent equable pressure they were cured without "—ED.

such as rose oil, alum, sumach and the like, together with emollients, poppies, linseed, etc. Their religion forbidding "the examination of women by the opposite sex" gynecological surgery found no place or favor with the celebrated physicians among the Mohammedans.

The nineteenth century dawned upon Rėcamier, Lisfranc and Astruc in France, Denman and John Clark in England, pushing gynecological research, and we get a glimpse of a contention that has continued *sub lite* and which is made the subject in part of the present paper, viz.: "Whether the local disorder is the cause or effect of the constitutional derangement, and whether as a consequence, treatment should be primarily directed to the one or the other?" The French holding the local disorder and the English the constitutional derangement precedent and causative. The consequence was natural. The French physicians are found in the lead in local treatment and topical application. In 1826, we see Guilbert recommended leeches to the cervix; in 1828, Lair, the use of the sound; in 1832, Melier, injections into the cervix; and Astruc, into the body of the womb; Recamier, the use of the curette; while Sir James Simpson, of Edinburgh, and Dr. Bennet, of London, were equally industrious in the advocacy of their views as to the constitutional nature of these troubles. After referring to the impetus given to gynecological surgery by such men as Simpson, Wells, Brown and Clay, in Great Britain; Simons, Esmarch, Ulrich, Hegar and Spiegelburg, of Germany, and of Sims, Atlee, Emmett, Bozeman, Peaslee, Dunlap, Agnew and Kimball in the United States, in the operation for ovariotomy, cure of ruptured perineum, vesico-vaginal fistula, constriction of cervix, prolapsus, etc., Prof. Thoams adds: "Professional opinion in their favor has of late years, like a pendulum, swung too far in one direction, gone to an extreme in the other." Continuing, he says: "The excessive surgical tendency of many leading gynecologists of our day is a matter to be deplored by all who wish well to gynecology. Every practitioner must often have seen cases in which pelvic peritonitis or cellulitis has arisen from an incision of the neck of the uterus, or some similar procedure, in which the patient is for weeks confined to the bed, and in which he is forced to doubt the necessity for the surgical resource which has been productive of the evil."

These words, true, as they most certainly were twenty years ago, when you could count on your fingers all the gynecological surgeons in this country who could be induced to resort to these extreme measures, with how much more emphasis do they hold now, when every little cross-roads tyro is ready, willing and waiting to make a laparotomy or do an ovariotomy. My observation of these operative procedures calls for and justifies words of reprobation much stronger than those used by Dr. Thomas. I may be permitted to express the belief that I appreciate these operations to the full for all that is in them. That in instances where called for, they mitigate pain, relieve suffering, and even prolong life, I am prepared to admit. I do not forget how easy it is in view of their gravity, to underestimate the good they have done. When told that our countryman, Dr. McDowell, had performed an ovariotomy, one of, if not quite, the greatest surgeons of France, exclaimed "He ought to be indicted for manslaughter." Even where a grave operation is necessary, constitutional treatment is no less so. Believing, as I most certainly do, from experience in the last forty years, that many, if not most of these have their origin in a vitiated condition of the general system, and while the system remains unrelieved any operative interference will generally be of no value, and should be had recourse to only as a dernier resort when death is imminent and life a burden. General innervation, and by this term I mean the office work of the whole nervous system, deteriorated to an extent making it inadequate to supply the necessary stimulus to establish the needed inter-communication between, and co-ordination of the other systems, for the due performance of their functions with consequent vitiation of the secretions, imperfect hæmatosis, nutrition and assimilation, certainly an operation promises little.

It is believed, the rule should be—of course, there are exceptions—to exhaust the resources of medical treatment, to do all within therapeutical competence to heal the lesion, to get rid of abnormal growths, and to restore the system to health, before resorting to the knife. It has been my fortune, or misfortune, —my lot at all events, to see a great number and variety of these troubles. A lunatic hospital, the wards of which I have walked for a number of years, is not a bad place for their observation. Have had from twenty to thirty under treatment at a time, and this running through years. It is popularly supposed, and the supposition shared in by the more ignorant of the profession, that all insane women have some trouble or other of the pelvic viscera. Of course, denial is needless in such a presence. Still, if the object is to see troubles peculiar to the sex, worse places might be selected than a hospital for the insane. There is another feature about these hospital cases,—owing to their excitement and lack of intelligence to appreciate their situation under surgical treatment, so as to act in such way as to favor recovery, the trouble they give and the care they require,—surgical interference is deferred and not had recourse to only as a dernier resort. Therefore medicinal treatment, constitutional and topical, is relied on.

It is not the purpose to inflict upon this body in this connection any dry details or statistics. I do not know what you may think of statistics. I regard cooked statistics, and they are generally cooked, as a nuisance. I believe the fellow was about right who said, "Nothing lies like facts except figures." Contenting myself, therefore, with giving the general result of my experience, I beg to summarize with the following conclusions:

1. Operations in cases of fistulæ of various kinds and extent, being attended with little danger, should be resorted to at once.

2. Where graver operations are called for or contemplated, and cases in which they offer the only basis for radical cure, it should be considered whether the patient is in a condition to survive the operation, how long she would probably live without interference, and in what comfort. Operations involving laparotomy are of course justified in certain circumstances, but not until the diagnosis is plainly made out by tapping, aspiration, or even explorative incision. The cases are not few in which such tentative procedure is called for, it being impossible to tell whether the operation is justified or not until such incision is made, and even then should be abandoned if complications in the shape of adhesion to important pelvic and abdominal viscera make the chances against the life of the patient.

Operative measures in cervicitis and endometritis, metritis and the thousand and one cuttings, burnings, borings, scrapings, curettings, kolpocleisis, elytroplasty, elytrorraphy, episiorraphy, and all this line of treatment, trusted to in place of medicinal treatment, topical application, conservative appliances, is bebelieved a great evil under the sun;—not that some of them may not occasionally be useful, but constitutional treatment is preferable, and is generally all that is needed.

These are but jottings in line of suggestion.

I beg to state a few things I have seen:—Some twenty years ago, in a lady, age about forty, a tumor, diagnosed as uterine interstitial fibroid, rendering micturition difficult, occluding the vagina so as to prevent insertion of finger. This patient was sent to Atlanta for an operation. Diagnosis was confirmed by Drs. Logan and Westmoreland, who, however, refused to operate, advising her to go to Marion Sims of New York. Refusing to go North, she returned to Texas, and came again into my hands for treatment. By application of glycerole of tannin the tumor, of whatever kind it may have been, disappeared, and she, already the mother of several children, became a mother again; is still living, in usual health at an advanced age.

Another case—one of complete procidentia: Organ enlarged to three times the normal size. As dry as a piece of parchment. Had been operated on twenty years before, Dr. Stone, of New Orleans, performing the operation, kolpocleisis. By depleting organ in usual way, returning to pelvic cavity and having patient wear Gold Globe pessary, in two years the cure was complete.

Another case: I was present at a laparotomy for ovarian cyst, involving all the pelvic viscera by adhesion, and as I recollect, the omentum as well. This was ascertained before the tumor was removed. Should the operation have been performed, even after the explorative incision? I thought then, and I think yet, the fluid drawn off, the wound should have been closed and the operation left alone, but it was completed and the woman died in forty-eight hours. By aspiration or tapping the woman might have lived on most probably for some years in tolerable comfort. The operation was performed *secundum artem* by one of the most distinguished surgeons of the South.

Another case: Was called to see a lady with some similar trouble on whom it was decided to operate. I was satisfied from her enfeebled condition and peculiar make-up that she would not survive the operation. Accordingly I advised against it. She was placed on supporting treatment. It has been three years since. She is, I learn, in tolerable health, well enough to cook a meal readily when she has no cook, entertains, and enjoys the companionship of her friends.

A word to close—a word the least agreeable and amiable of all. It is to be feared that many of the younger members of the profession are attracted by the eclat and dazzled by the glamour of these operations. Beware. All is not gold that glitters—all is not glory that sounds big. It is a small matter to cut open a woman's belly—anyone can remove an ovary that can spay a pig.

Nobody knows better than I do that it is unpopular to use such language. I am too old to care much for popularity. Believing with the foremost philosopher of modern times that "our thoughts are our children we may not needlessly let die," I say what I think, and in the familiar words of an unfamiliar language:

"Vive valeque—Siquid novisti rectius istis
Condidus imperti; Si non, his utere mecum."

